# Effect of vitamin K2 administration on depression status in patients with polycystic ovary syndrome: a randomized clinical trial

**DOI:** 10.1186/s12905-022-01825-8

**Published:** 2022-07-26

**Authors:** Firoozeh Tarkesh, Bahia Namavar Jahromi, Najmeh Hejazi, Golazin Hoseini

**Affiliations:** 1 Gastroenterohepatology Research Center, Shiraz University of Medical Sciences, Shiraz, Iran; 2 Infertility Research Center, Shiraz University of Medical Sciences, Shiraz, Iran; 3 Department of OB-GYN, School of Medicine, Shiraz University of Medical Sciences, Shiraz, Iran; 4 Nutrition Research Center, Department of Clinical Nutrition, School of Nutrition and Food Sciences, Shiraz University of Medical Sciences, Shiraz, Iran; 5 Student Research Committee, Clinical Nutrition Department, School of Nutrition and Food Sciences, Shiraz University of Medical Sciences, Shiraz, Iran

**Keywords:** Vitamin K2, Polycystic ovary syndrome, Depression

## Abstract

**Background:**

Patients with Polycystic ovary syndrome (PCOS) are predisposed to the development of several mental comorbidities such as depression. According to several studies, PCOS can be managed by improving insulin sensitivity. The insulin-sensitizing effect of vitamin K has been reported in recent studies. Therefore, in the current trial, we assessed the effect of administrating vitamin K2 (Menaquinone-7) on depression status in women afflicted with PCOS.

**Methods:**

Eighty-four PCOS women were allocated into the intervention and comparison groups; the intervention group (n = 42) administered 90 µg/day Menaquinone-7, and the comparison group (n = 42) consumed placebo capsules (containing avesil) for 8 weeks. In this randomized, double blind, placebo-controlled clinical trial, depression status was measured by BECK depression inventory-II (BDI-II) before and after 8 weeks of intervention.

**Results:**

Consumption of Menaquinone-7 in comparison with the placebo capsules significantly improved depression status (*P* = 0.012).

**Conclusion:**

This clinical study reported the advantageous effect of Menaquinone-7 administration on depression status in PCOS patients.

*Trial registration* The present study was registered at http://www.IRCT.ir on 06/06/2018 (registration number: IRCT20170916036204N5).

## Background

Polycystic ovary syndrome (PCOS) is a frequent endocrinopathy with the prevalence of 9–18% among women of reproductive age [1–3].

This syndrome, according to the Rotterdam criteria, is characterized by the presence of at least 2 features of the following characteristics: 1. polycystic ovaries 2. oligo or anovulation 3. biochemical and/or clinical symptoms of hyperandrogenism [4, 5]. In addition to these features, an array of feminine problems including hirsutism, acne and scalp hair thinning and infertility can prone PCOS patients to several mental disorders, namely anxiety, bipolar disorder, depression, and eating disorders [6]. Also, PCOS can increase the risk of pregnancy complications [7].

Although the root cause of PCOS is not completely comprehended, insulin resistance (IR) has been reported as the leading cause of PCOS according to a number of studies [8]. IR in these patients can create hyperinsulinemia which increases serum free androgen levels through increasing the hepatic generation of sex hormone binding globulin (SHBG), and androgen production by ovarian theca cells [9].

Changing life-style is the first-line strategy for managing PCOS. Even small changes in diet and physical activity can improve insulin resistance which leads to ameliorate the complications of PCOS [6, 10]. Also, diet restriction and fasting might improve the metabolic imbalance typical of PCOS [11, 12].

The use of insulin-sensitizers has gained growing attention because of their significant efficiency on PCOS [13, 14]. Recently, a number of studies reported the insulin-sensitizing effect of vitamin K [15]. Based on these studies, vitamin K can enhance insulin sensitivity through inducing adiponectin expression [16].

Phylloquinone (vitamin K1) and menaquinone (vitamin K2) are two natural forms of vitamin K.

The current recommendations for vitamin K dietary intake are according to the daily dose which is vital for preventing bleeding [17]; in normal situations such as normal pregnancy, there is no need for vitamin K supplementation [18].

It has been demonstrated that vitamin K can be mainly found in the form of menaquinone in brain [19]. Gancheva et al. investigated the effect of vitamin K on depression for the first time. In this animal study, menaquinone-7 administration prevented the anxiety and depression progression in rats suffering from metabolic syndrome [20].

It should be noted that no paper has reported the effect of menaquinone-7 on depression status in PCOS patients. Therefore, the effect of menaquinone-7 administration on depression status in PCOS patients has been investigated in this clinical study.

## Materials and methods

### Participants

Between July and September 2016, the present study was performed on 84 women who were referred to the infertility clinic of Ghadir Mother & Child Hospital in Shiraz, Iran.

PCOS women aged 18–40 years who were diagnosed based on the Rotterdam criteria were eligible to participate in this study [21]. Exclusion criteria consisted of following specific physical activity program or diet for three months prior to the beginning of the study, administrating medications or supplements that were likely to affect glucose metabolism, bone metabolism, lipid profile, and ovarian function, and using any antibiotics and anticoagulant drugs such as warfarin for 3 months prior to the study. Pregnant/lactating individuals, and patients with Cushing syndrome, thyroid disorders, congenital adrenal hyperplasia, hypertension, and diabetes mellitus were also excluded from participating in this study.

This trial was accomplished in accordance with the Declaration of Helsinki and good clinical practice guidelines. The local Ethics Committee of Shiraz University of Medical Sciences reviewed and approved the protocol of this study (IR.SUMS.REC.1397.102). This trial was recorded in the Iranian registry of clinical trials (first registration date: 04-06-2018**,** registration number**:** IRCT20170916036204N5).

The protocol of this study was described for all the participants; therefore, they signed the inform consent consciously.

Thirty-seven women were calculated to be in each group based on the decreased level of serum glucose in a previous study [22] with 0.05 significance level and power of 80%. Eventually, 42 women were determined in each study arm considering a 15% dropout rate.

### Study design

**Fig. 1:**
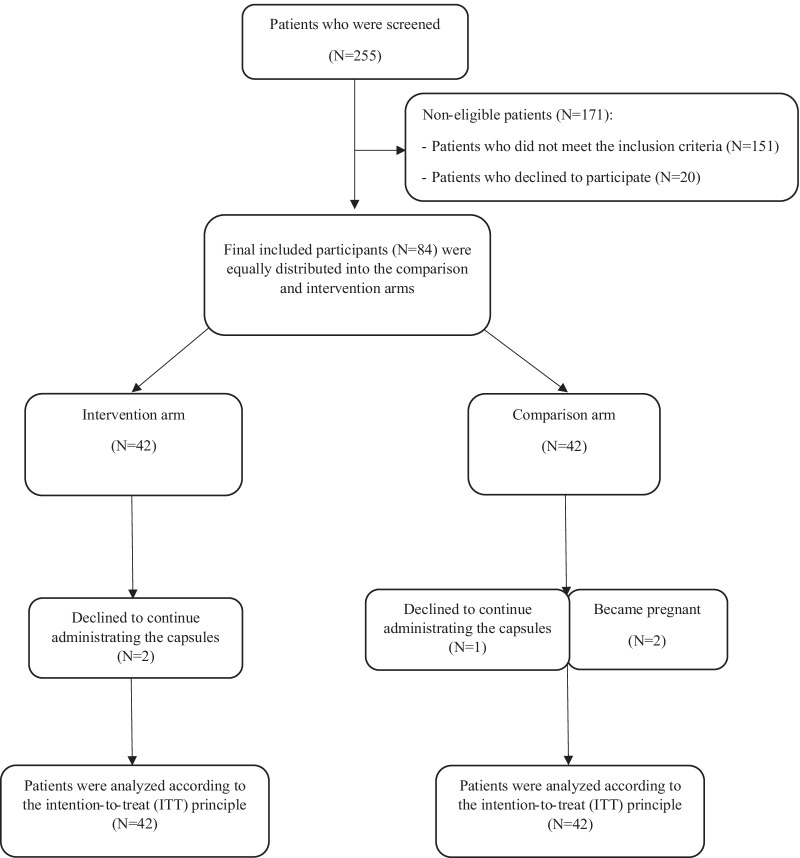
Flowchart of the participants

Totally, 255 patients with PCOS were screened and eighty-four subjects participated in this randomized, double-blind, placebo-controlled clinical trial, as shown in the consort flow diagram (Fig. 1).

All the eligible patients were evenly randomized into two arms of comparison and intervention by block randomization. This process was performed with a fixed block size of 4 using random allocation software [23]. Subjects in the intervention group administered one capsule (90 µg of Menaquinone-7), and participants in the comparison arm consumed a placebo capsule (avesil) on a daily basis for 8 weeks.

All the capsules were equal regarding size (size 0), color (white), aroma and weight. Arian Salamat Sina Company (Tehran, Iran) provided all these capsules specifically for the present trial in order to blind the patients, investigators and infertility clinic staff for group assignment. A leaflet including some PCOS dietary recommendations was given to each participant. We asked the participants not to alter their usual diet and physical activity level, and report any side-effect within the 8-week study period.

A daily text message was forwarded to each patient’s contact number to evaluate participants’ adherence. Moreover, the patients were asked to return their capsule packets after the intervention period, and if they had consumed 90 percent of the capsules, we assumed them adherent.

Three-day dietary records were completed by participants before and after this trial. We computed the patients’ daily energy and intakes of macronutrients (carbohydrate, fat and protein) and micronutrients (vitamin K and vitamin D) by means of Nutritionist 4 software (first data bank, San Bruno, CA, USA) that was adjusted for Persian foods [24]. In addition, the level of physical activity was evaluated through completing the validated version of International Physical Activity Questionnaire (IPAQ) at baseline of the trial [25, 26].

### Measurement of parameters

#### Anthropometry and depression assessments

We measured weight (to 0.1 kg) and height (to 0.1 cm) using Seca scales (Hamburg, Germany) for each patient after an overnight fast before and after this clinical study. Moreover, Body mass index (BMI) was calculated through dividing weight (kg) by height (m^2^). For assessing the intensity of depression, all participants completed BECK depression inventory-II (BDI-II) at baseline and at the end of the study [27].

#### Blood sampling and vitamin K assessment

After 12 h of fasting, 7 ml of venous blood were collected before the trial. Blood samples were centrifuged at 2,000 g/min for 10 min, and the separated serum samples were frozen at − 80 °C.

Serum level of vitamin K was measured by ELISA kit (ZellBio, Catalog No. RK00737).

### Statistical analysis

All of the statistical analyses were done by means of statistical package for the social sciences, version 21 (SPSS, Inc. Chicago, USA). The parametric information was reported as mean ± SD and the non-parametric information was shown as median (IQR).

We performed Levene’s and Kolmogorov–Smirnov tests for checking homoscedasticity of data and normal/abnormal distribution. For comparing the variables between study groups, we used an independent sample t-test to compare the normally distributed data, and the Mann–Whitney U-test for skewed data. A pair t-test was performed for comparing the parametric information of depression status in each study arm before and after the intervention.

## Results

Eighty-four PCOS women were eligible to participate in this study. During this study, five patients dropped out; two participants in the MK-7 arm were reluctant to administer the capsules, and two women became pregnant and a participant was lost to follow-up in the comparison arm (Fig. 1).

Almost 90% of the participants in each study arm completely followed the protocol of this study. The participants did not report any adverse effect during this 8-week trial.

Baseline data related to the participants can be observed in Table 1; there were not any significant differences between the intervention and comparison arms regarding anthropometric measurements, physical activity level, age, fasting blood sugar, serum vitamin K2 and depression status.

**Table 1:** Baseline data in the intervention and comparison arms

Variables	Intervention arm (n = 42)	Comparison arm (n = 42)	Comparison between groups (*P*-value)*
Age (y)	28 (26–30)	27 (24–28)	0.71
Weight (kg)	68.02 ± 11.28	70.47 ± 14.08	0.39
Body Mass Index (kg/m^2^)	25.93 ± 4.18	27.31 ± 4.78	0.16
Physical activity level (MET-min/week)	594 (198–1282.5)	462 (231–997.5)	0.5
Fasting blood sugar (mg/dl)	90.32 ± 7.52	89.19 ± 8.5	0.52
Serum level of vitamin K (ng/ml)	436.95 (400–495.7)	434.3 (401.2–492.02)	0.85
Depression status	16.9 ± 7.918	13.78 ± 9.05	0.1

*Parametric data were analyzed by independent samples *t* test, and non-parametric data were Mann–Whitney U test

*P*-values less than 0.05 were considered as the significant level of differences

Information is shown as mean ± SD or median (IQR)

Table 2 shows depression status before and after this 8-week trial in all participants.

**Table 2:** Comparison of depression status in the participated at baseline and at the end of the intervention

Variable	Vitamin-administered arm (n = 42)			Placebo-administered arm (n = 42)			*P*-value**
	Before the intervention	At the end of the intervention	*P*-value*	Before the intervention	At the end of the intervention	*P*-value*	
Depression status	16.9 ± 7.918	14.975 ± 8.3	0.013	13.78 ± 9.05	14.02 ± 9.54	0.84	0.012

*Data were analyzed by paired samples *t* test

**Data were analyzed by independent samples *t* test

*P*-values less than 0.05 were presumed as the significant level of differences

Information is shown as mean ± SD

Consumption of MK-7 in comparison with the placebo capsules significantly improved depression status (*P* = 0.012).

Regarding reported macro-nutrient and micro-nutrient intakes, there were no significant differences between the intervention and comparison groups during the study.

## Discussion

To our knowledge, this clinical study is the first trial that has investigated the effect of vitamin K on depression status in PCOS patients. The findings of this trial reported that an eight-week administration of MK-7 significantly improved depression status in PCOS women.

A number of previous studies have shown that PCOS patients may experience depression [28]. Factors such as inflammation and neurotransmitter dysfunction were discussed to be involved in depression pathogenesis [29].

The NF-KB pathway can be regulated by vitamin K, as an anti-inflammatory agent, which can suppress the expression of inflammatory cytokines [30]. Moreover, lack of vitamin K in brain can increase ceramides; as the concentration of brain ceramides increases, inflammatory cytokines namely IL-2 and IL-6, and reactive oxygen species (ROS) production can be increased [30–32].

In addition, several studies have shown that vitamin K can enhance nerve growth factor (NGF) and brain-derived neurotrophic factor (BDNF) through activating protein kinase A; it has been hypothesized that a decline in BDNF and NGF in hippocampus can be associated with depressive behaviors [31, 32]. In line with the mentioned results, Silvia M Gancheva et al. [20] reported that the 10-week administration of vitamin K2 by oral gavage prevented the development of depression in rats afflicted with metabolic syndrome. Also, Turker et al., found out that individuals with atrial fibrillation administrating warfarin, a vitamin K antagonist, were more likely to suffer from depression compared to patients with atrial fibrillation consuming dabigatran, a non-vitamin K anticoagulant [33].

Along with these findings, the current study has shown the anti-depressive effect of vitamin K2 in PCOS women. On the contrary, Rubio-López et al. reported a positive association between depressive symptoms and the dietary intake of vitamin K in Spanish children [34]. The contradiction between these results might be because of the difference in depression questionnaires (BECK vs CES-DS) and target subjects (PCOS women vs children).

A few studies hypothesized that insulin resistance (IR) might be related to depression in women with PCOS [35]. Regarding the impact of vitamin K on the metabolism of glucose, this fat-soluble vitamin can improve insulin sensitivity by increasing adiponectin expression [36]. Plus, osteocalcin, a vitamin K-dependent protein, induces insulin secretion by affecting pancreatic β-cells [36–38]. Having said all that, in our previous study [24], fasting blood sugar was not significantly affected by 8 weeks of MK-7 consumption compared to administrating placebo capsules in PCOS patients. In line with the mentioned study, Shahdadian et al. [39] in a systematic review and meta-analysis reported that vitamin K administration could not significantly affect glycemic control within healthy individuals. In another study [40], after 4 weeks of vitamin k2 administration by healthy young men, insulin sensitivity increased but fasting blood glucose did not significantly change. Also, Knapen et al. did not observe any significant alteration in fasting blood sugar after three years of MK-7 supplementation in postmenopausal women [41]. In our previous study [24], all the participants had normal serum vitamin K level at baseline; it can be hypothesized that vitamin K administration may have greater effects on fasting blood sugar in vitamin K deficient individuals.

Based on our knowledge, this paper has investigated the effect of vitamin K on depression status for the first time; this can be considered as a study strength. Lack of funding was the limitation of our study as we could not measure serum levels of osteocalcin, adiponectin, and inflammatory markers to better interpret the potential mechanisms led to improve our patients’ depression status. Further studies of the issue are still required; clinical studies with longer intervention periods and larger sample sizes are needed for confirming the anti-depressant effect of vitamin K in PCOS women.

## Conclusion

This clinical study reported the possible anti-depressant effect of vitamin K2 in PCOS women for the first time. However, additional clinical studies with longer intervention periods and larger sample sizes should be performed to confirm the anti-depressant effect of vitamin K in PCOS women.

## Data Availability

The datasets used and analyzed during the current study are available from the corresponding author on reasonable request.

## Abbreviations

PCOS: Polycystic ovary syndrome
IR: Insulin resistance
SHBG: Sex hormone binding globulin
IPAQ: International Physical Activity Questionnaire
BMI: Body mass index
BDI-II: BECK depression inventory-II
ROS: Reactive oxygen species
NGF: Nerve growth factor
BDNF: Brain-derived neurotrophic factor
